# Supplementary figures and images for: Chromosome length is not the sole determinant of sexually dimorphic crossover rates during mammalian meiosis: Insights from genetically diverse mouse strains
Source: bioRxiv. 2025 Dec 22:2025.12.19.695521. Preprint. [Version 1] doi: 10.64898/2025.12.19.695521 (PMC12776159; doi:10.64898/2025.12.19.695521)

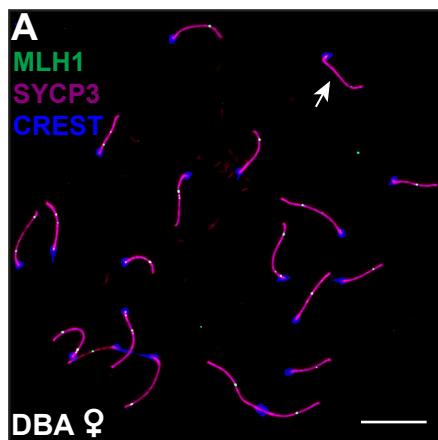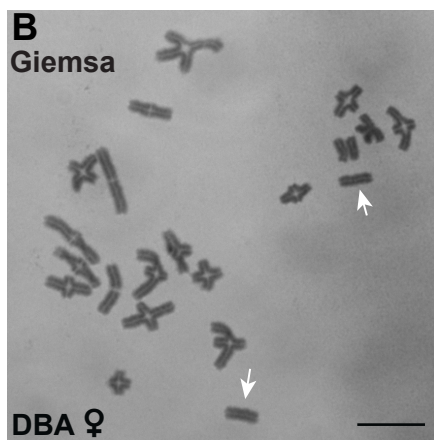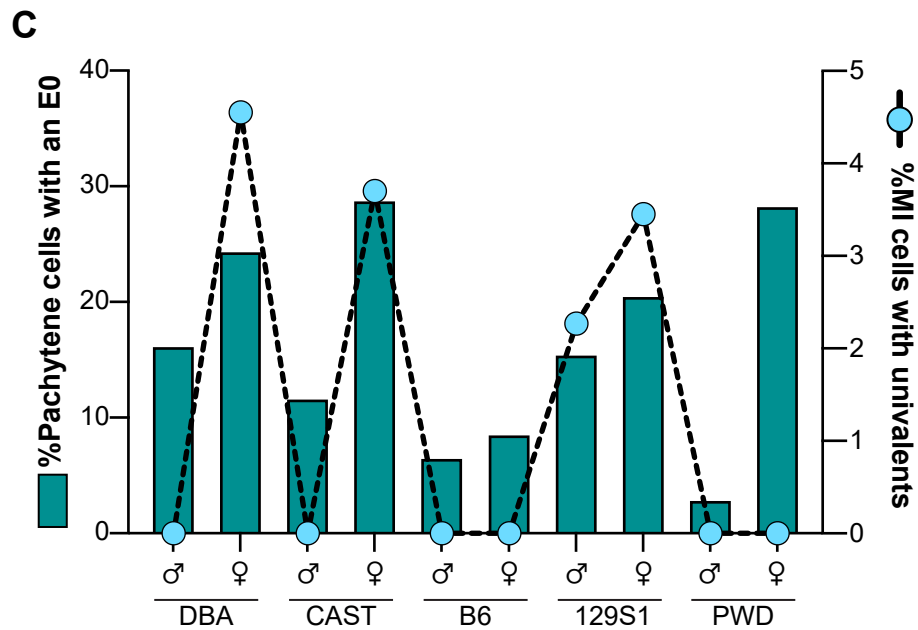

Supplement: Supplement 2 — (A) Representative immunofluorescence image of a DBA oocyte in which one chromosome pair failed to make a class I CO (E0, white arrow). (B) Representative Giemsa-stained DBA oocyte chiasmata spread showing unpaired homologous chromosomes (univalents, white arrow). (C) Percentage of pachytene cells with at least one E0 SC (dark teal bars, left axis) and percentage of metaphase I cells with univalents (light teal circles, right axis). Although chromosome pairs without a class I CO—predicted to result in unpaired chromosomes at metaphase I that likely mis-segregate—are relatively common, the actual incidence of metaphase I cells with univalents is strikingly low across strains and sexes, indicating that in many meiotic cells, the obligate CO is made via the class II pathway. Scale bars, 10 µm. [file media-2.pdf]

**A**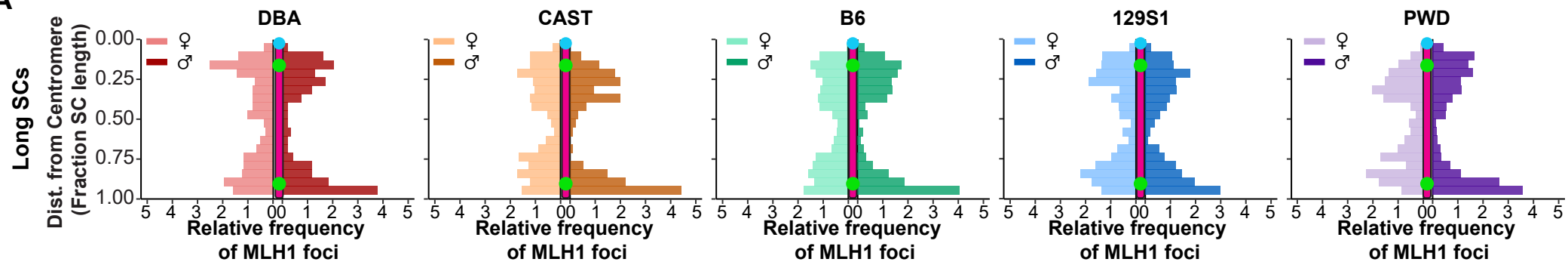**B**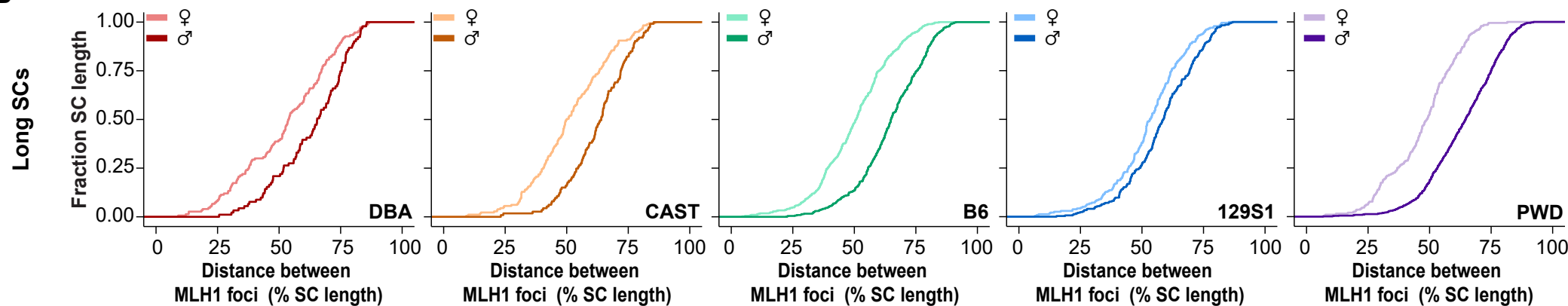**C**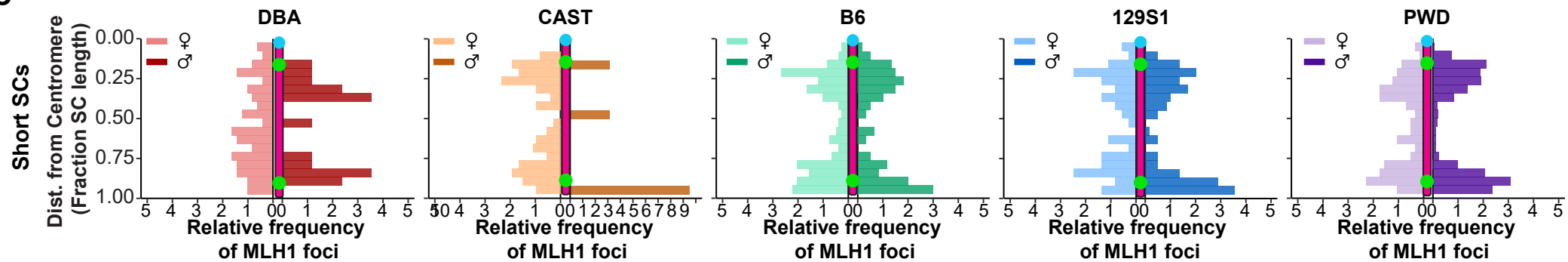**D**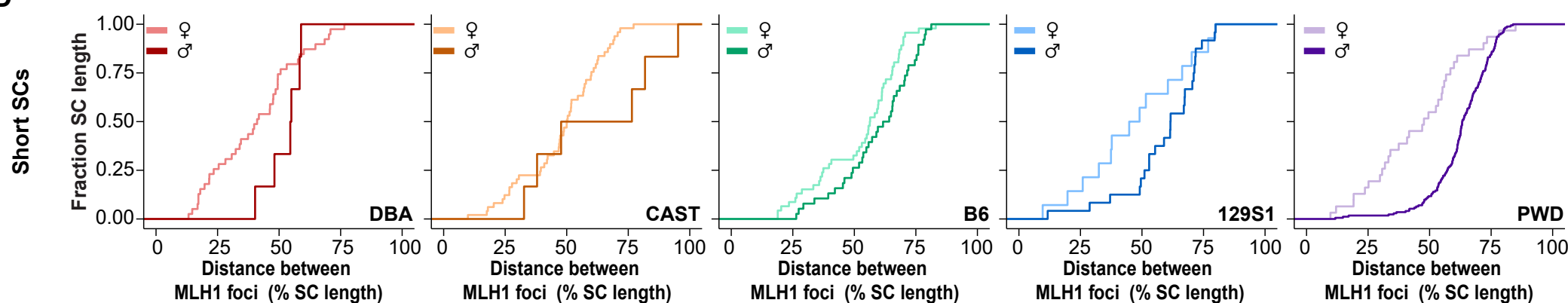

Supplement: Supplement 4 — Expanded analysis from Fig. 3 examining CO interference on the longest (A-B) and shortest (C-D) five SCs per cell. Histograms (A, C) show the relative frequency of MLH1 foci at terminal SC ends (0.0–0.25 and 0.75–1.0). The y-axis represents fractional SC length from centromere (0.0) to distal telomere (1.0). (A) For long SCs, males (dark bars) had significantly more terminally placed MLH1 foci than females (light bars; Fisher’s exact test): 74.43% vs. 65.15% in DBA (OR = 0.64, p < 0.05), 69.44% vs. 58.05% in CAST (OR = 0.61, p < 0.05), 72.80% vs. 59.05% in B6 (OR = 0.54, p < 0.0001), and 74.61% vs. 55.60% in PWD (OR = 0.35, p < 0.0001), but not in 129S1 (66.92% vs. 63.14%, p = 0.3). (C) Among short SCs, only PWD males showed greater terminal placement (75.72% vs. 55.56%; OR = 0.40, p < 0.0001). No significant sex differences were observed in other strains (male vs. female: DBA, 56.25% vs. 52.17%, p = 0.79; CAST, 83.33% vs. 61.59%, p = 0.41; B6, 68.42% vs. 66.19%, p = 0.71; 129S1, 69.64% vs. 64.29%, p = 0.52). (B) ECDF graphs of standardized inter-focus distance (%SC length) for long SCs show significantly greater distances in males for all strains (Kolmogorov-Smirnov: DBA, KS = 0.32, p < 0.01; CAST, KS = 0.35, p < 0.0001; B6, KS = 0.40, p < 0.0001; 129S1, KS = 0.21, p < 0.01; PWD, KS = 0.43, p < 0.0001). (D) For short SCs, only PWD males showed significantly greater inter-focus distance than females (KS = 0.49, p < 0.001) [file media-4.pdf]

# Mid/Late Pachynema

MSH4 MLH3 SYCP3

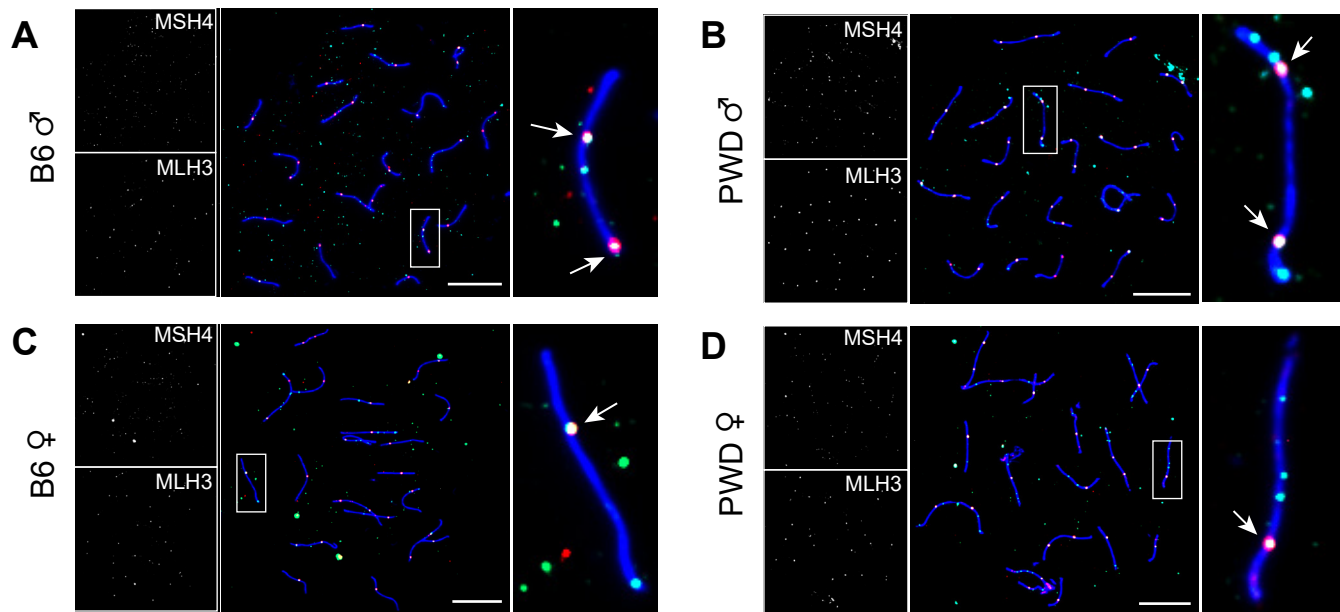

E

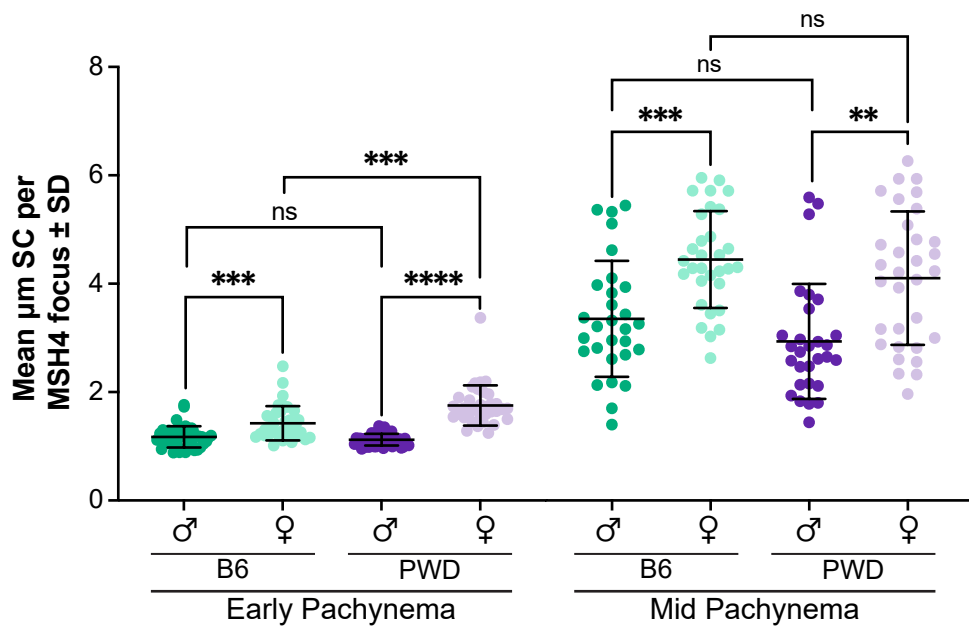

F

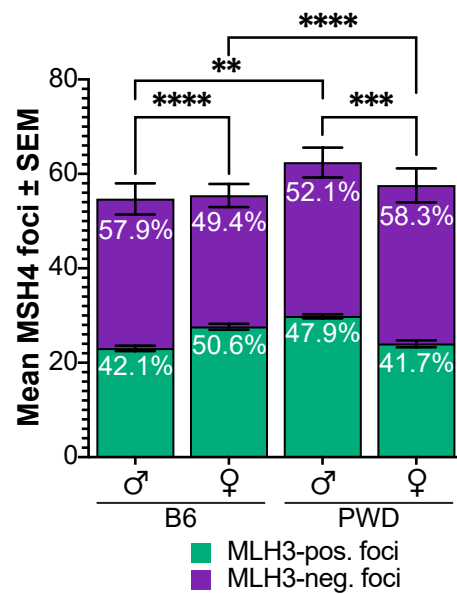

Supplement: Supplement 5 — Representative images of mid-pachytene spermatocytes (A-B) and oocytes (C-D) from B6 (A, C) and PWD (B, D) mice stained for MSH4 (green), MLH3 (red), and SYCP3 (blue). (E) Comparison of mean microns of SC per MSH4 focus for B6 and PWD males and females at early (F = 45.11, p<0.0001) and mid-pachynema (F = 12.46, p<0.0001). Significant sex differences in mean microns/focus were observed in B6 (early: t = 4.14; mid: t = 4.29) and PWD (early: t = 9.91; mid: t = 3.98). Significant strain differences were only observed between females at early pachynema (t = 4.05). (F) The proportion of MSH4 foci colocalized with MLH3 at mid-pachynema showed significant sex differences (B6: X2 = 23.1; PWD: X2 = 13.9) and strain differences (males: X2 = 11.1; females: X2 = 27.5). Asterisks denote significant differences: **** p < 0.0001, *** p < 0.001, ** p < 0.01 (Games-Howell for E; Bonferroni for F). Error bars represent SD (E) or SEM (F). Scale bars represent 10 µm. [file media-5.pdf]

**A**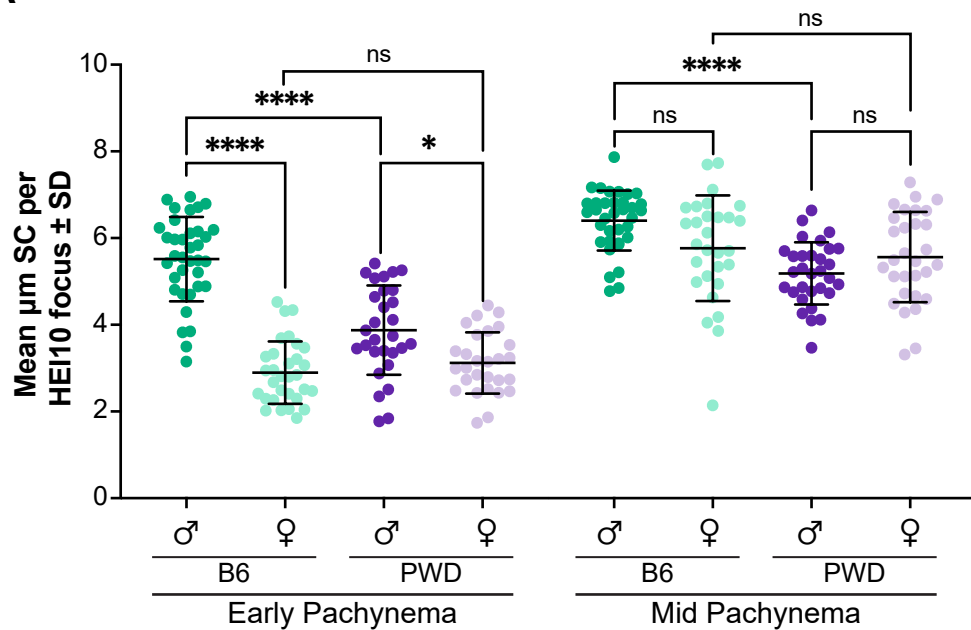**B**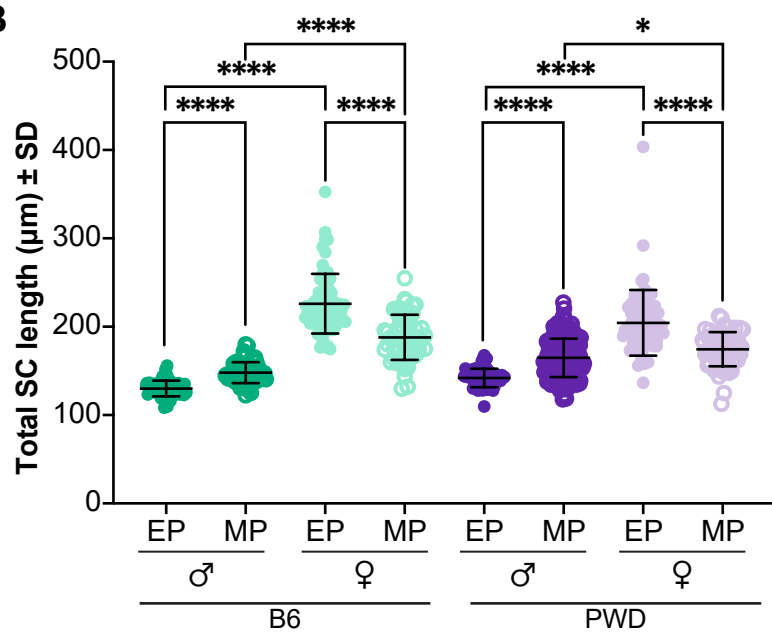**C**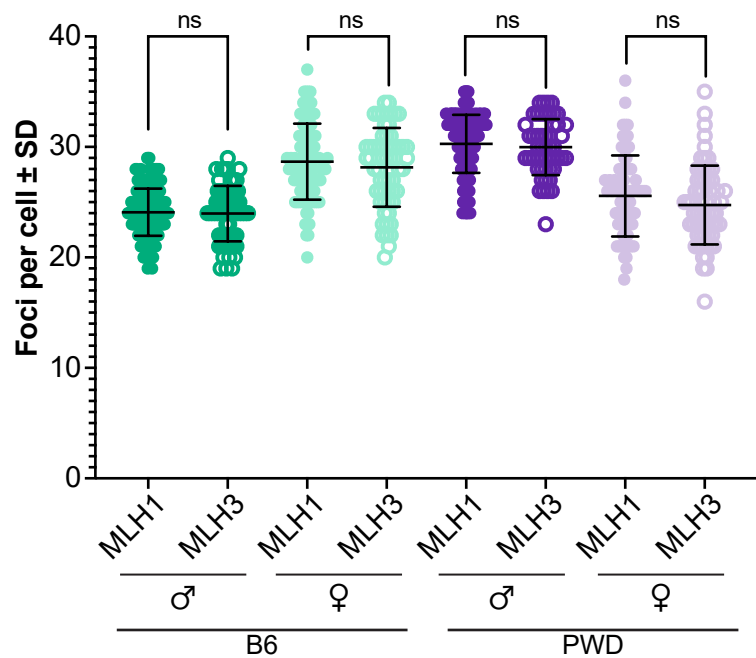

Supplement: Supplement 6 — (A) Mean microns of SC per HEI10 focus for B6 and PWD males and females at early and mid-pachynema. Significant sex differences in mean microns/focus were observed only at early pachynema in B6 (early: t = 12.6; mid: t = 2.5) and PWD (early: t = 3.25; mid: t = 1.64). Significant strain differences were only observed between males (early: t = 6.54; mid: t = 7.01). (B) Total SC length at pachynema (F = 134.3, p<0.0001) is shorter at early (closed circles) versus mid (open circles) stages for B6 (t = 10.09) and PWD (t = 11.42) males, but longer at early pachynema for B6 (t = 7.22) and PWD (t = 5.61) females. Total SC length is consistently greater in females than males for B6 (early: t = 22.66; mid: t = 11.02) and PWD (early: t = 12.71; mid: t = 3.42). (C) For B6 and PWD male and female mid-pachytene cells, mean MLH1 foci number (closed circles) does not differ significantly from mean MLH3 foci number (open circles). Asterisks denote significant differences: **** p < 0.0001, * p < 0.05 (Games-Howell post-hoc). Error bars represent SD. [file media-6.pdf]
