# Supplementary material for: Chromosome length is not the sole determinant of sexually dimorphic crossover rates during mammalian meiosis: Insights from genetically diverse mouse strains
Source: bioRxiv. 2025 Dec 22:2025.12.19.695521. Preprint. [Version 1] doi: 10.64898/2025.12.19.695521 (PMC12776159; doi:10.64898/2025.12.19.695521)
Supplement: Supplement 3 — (A) Effect of SC length on the predicted probability of an SC having more than one MLH1 focus for DBA (red), CAST (orange), B6 (green), 129S1 blue), and PWD (purple) males (dark) and females (light). Logistic regression analysis and significance are in Table S2. (B) Each SC per nucleus was classified as long (5 longest), short (5 shortest), or medium (9-10 intermediate-length). The percentage of SCs in each length category without an MLH1 focus (light teal), with 1 focus (teal), or with multiple foci (dark teal) is plotted for each sex and strain. (C) Logistic regression analysis of 1231 the effects of sex, strain, and SC length (continuous variable) on the likelihood of an SC having multiple MLH1 foci (B6 female as reference). This model predicted the likelihood of multiple MLH1 foci for SCs of three lengths: 5, 10, and 15 µm. Odds ratios below 1.0 indicate higher likelihood in males. Note that 15 µm SCs were exceptionally rare in males (absent in DBA males). [file media-3.pdf]

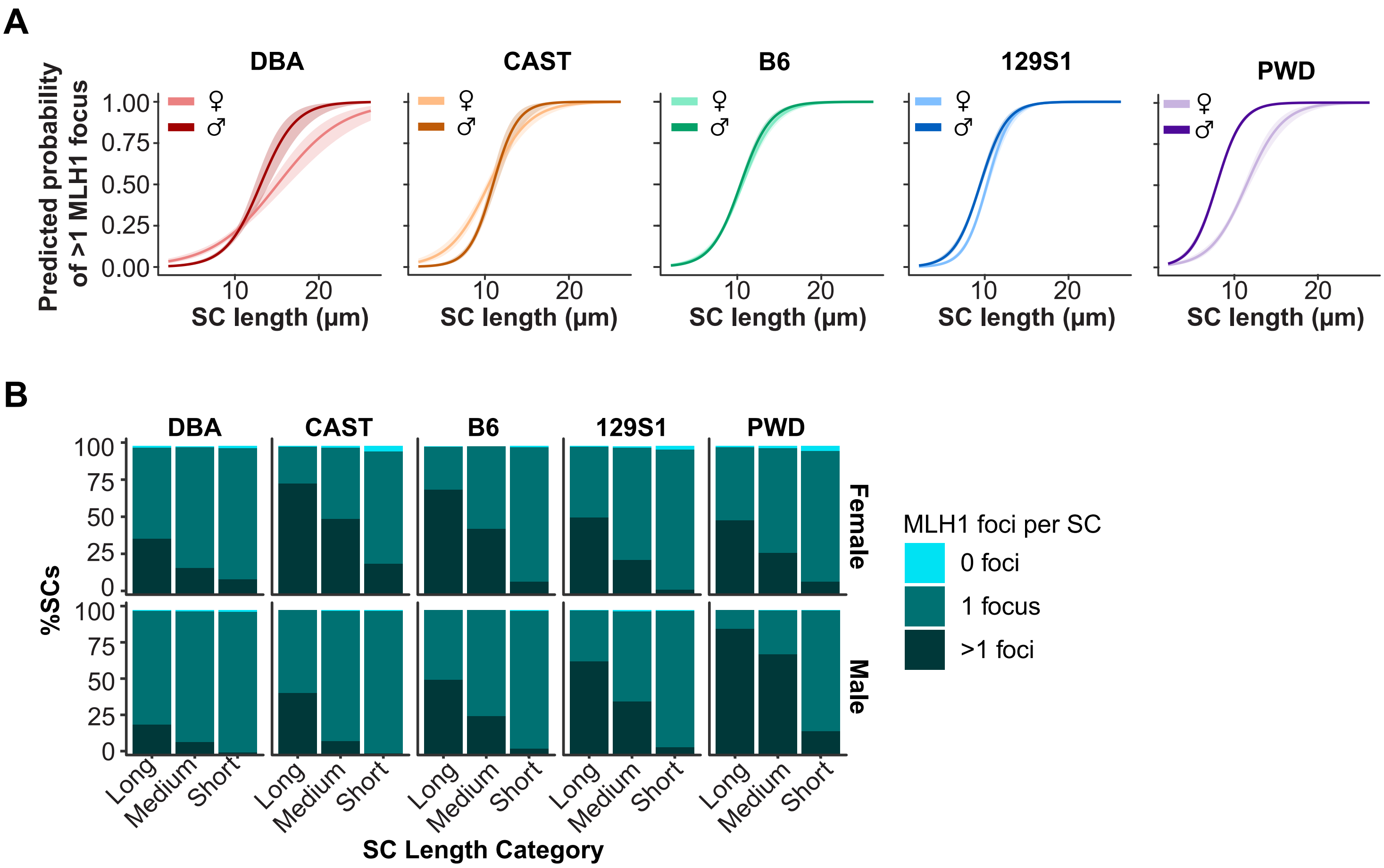

C

SC Length (µm)	Strain	Odds Ratio	SE	z-ratio	p-value
	DBA	3.589	0.93	4.96	<0.0001
	CAST	6.605	2.22	5.62	<0.0001
5 µm	B6	1.165	0.22	0.83	0.409
	129S1	0.381	0.09	-4.16	<0.0001
	PWD	0.362	0.07	-5.12	<0.0001
	DBA	1.184	0.14	1.47	0.142
	CAST	1.670	0.23	3.78	<0.001
10 µm	B6	0.912	0.07	-1.26	0.207
	129S1	0.583	0.06	-5.01	<0.0001
	PWD	0.129	0.01	-23.39	<0.0001
	DBA	0.391	0.12	-2.95	<0.01
	CAST	0.422	0.18	-2.07	<0.05
15 µm	B6	0.714	0.16	-1.54	0.124
	129S1	0.892	0.31	-0.33	0.745
	PWD	0.046	0.01	-11.88	<0.0001
