# Supplementary material for: Chromosome length is not the sole determinant of sexually dimorphic crossover rates during mammalian meiosis: Insights from genetically diverse mouse strains
Source: bioRxiv. 2025 Dec 22:2025.12.19.695521. Preprint. [Version 1] doi: 10.64898/2025.12.19.695521 (PMC12776159; doi:10.64898/2025.12.19.695521)
Supplement: Supplement 7 — Summary of Brown-Forsythe ANOVA test and subsequent Games-Howell’s multiple comparison test for significant differences in mean SC length, MLH1 foci number, microns SC per MLH1 focus, and chiasmata number between all sexes and strains. [file media-7.pdf]

	DBA/2J ♂	DBA/2J ♀	CAST/EiJ ♂	CAST/EiJ ♀	C57Bl/6J ♂	C57Bl/6J ♀	129S1/SvImJ ♂	129S1/SvImJ ♀	PWD/PhJ ♂	PWD/PhJ ♀
**N Animals (cells)**	3 (86)	3 (78)	3 (51)	3 (45)	7 (165)	4 (104)	3 (90)	3 (86)	7 (173)	3 (74)
**Mean SC length (µm) ± SEM**	149.2 ± 1.43	188.3 ± 3.00	149.3 ± 1.34	209.5 ± 5.14	156.6 ± 1.05	196.8 ± 2.39	156.2 ± 1.35	164.4 ± 1.97	168.3 ± 1.66	181.6 ± 2.32
**N Animals (cells)**	3 (86)	3 (94)	3 (51)	3 (45)	7 (200)	4 (104)	3 (90)	4 (132)	7 (203)	3 (74)
**Mean MLH1 foci ± SEM**	20.78 ± 0.17	24.31 ± 0.26	21.94 ± 0.22	30.16 ± 0.57	24.09 ± 0.15	28.66 ± 0.34	25.77 ± 0.27	25.05 ± 0.26	30.28 ± 0.18	25.57 ± 0.43
**N Animals (cells)**	3 (86)	3 (78)	3 (51)	3 (45)	7 (165)	4 (104)	3 (90)	3 (86)	7 (173)	3 (74)
**Mean microns SC per MLH1 focus ± SEM**	7.37 ± 0.07	8.27 ± 0.12	7.08 ± 0.07	7.45 ± 0.13	6.91 ± 0.05	7.46 ± 0.10	6.50 ± 0.07	6.95 ± 0.07	5.86 ± 0.05	7.58 ± 0.11
**N Animals (cells)**	3 (47)	9 (44)	3 (28)	9 (27)	3 (59)	4 (27)	3 (44)	6 (29)	3 (52)	11 (34)
**Mean chiasmata ± SEM**	20.78 ± 0.17	24.31 ± 0.26	21.94 ± 0.22	30.16 ± 0.57	24.09 ± 0.15	28.66 ± 0.34	25.77 ± 0.27	25.05 ± 0.26	30.28 ± 0.18	25.57 ± 0.43

Brown-Forsythe ANOVA test	***SC length***	***MLH1 foci***	***Microns SC/MLH1 focus***	***Chiasmata***
**F (DFn, DFd)**	74.2 (9.0, 365.0)	135.6 (9.0, 529.8)	77.75 (9.0, 603.2)	61.2 (9.0, 233.8)
**P value**	<0.0001	< 0.0001	<0.0001	<0.0001

Games-Howell's multiple comparisons test	***SC length***				***MLH1 foci***				***Microns SC/MLH1 focus***				***Chiasmata***			
**Comparison**	**Mean Diff.**	**t**	**DF**	**Adj.P Value**	**Mean Diff.**	**t**	**DF**	**Adj.P Value**	**Mean Diff.**	**t**	**DF**	**Adj.P Value**	**Mean Diff.**	**t**	**DF**	**Adj.P Value**
DBA/2J ♂ vs. DBA/2J ♀	-39.2	11.8	110.9	<0.0001	-3.5	11.6	157.4	<0.0001	-0.9	6.4	125.0	<0.0001	-1.6	3.2	59.0	0.0599
DBA/2J ♂ vs. CAST/EiJ ♂	-0.2	0.1	130.0	>0.9999	-1.2	4.2	104.6	0.0019	0.3	2.8	123.6	0.1350	-0.3	0.9	56.7	0.9956
DBA/2J ♂ vs. CAST/EiJ ♀	-60.3	11.3	50.9	<0.0001	-9.4	15.7	51.6	<0.0001	-0.1	0.5	69.8	>0.9999	-6.8	16.7	42.8	<0.0001
DBA/2J ♂ vs. C57Bl/6J ♂	-7.4	4.2	174.8	0.0018	-3.3	14.7	218.6	<0.0001	0.5	5.2	173.8	<0.0001	-2.0	6.6	104.0	<0.0001
DBA/2J ♂ vs. C57Bl/6J ♀	-47.7	17.1	164.4	<0.0001	-7.9	20.9	148.6	<0.0001	-0.1	0.7	180.1	0.9996	-5.8	12.8	38.8	<0.0001
DBA/2J ♂ vs. 129S1/SvImJ ♂	-7.1	3.6	172.8	0.0155	-5.0	15.8	148.2	<0.0001	0.9	9.0	172.3	<0.0001	-1.3	4.4	86.8	0.0012
DBA/2J ♂ vs. 129S1/SvImJ ♀	-15.2	6.2	155.2	<0.0001	-4.3	13.9	207.1	<0.0001	0.4	4.4	168.7	0.0008	-3.5	5.7	34.6	<0.0001
DBA/2J ♂ vs. PWD/PhJ ♂	-19.1	8.7	246.5	<0.0001	-9.5	38.3	257.9	<0.0001	1.5	17.2	173.3	<0.0001	-7.7	23.2	92.3	<0.0001
DBA/2J ♂ vs. PWD/PhJ ♀	-32.5	11.9	123.8	<0.0001	-4.8	10.5	95.1	<0.0001	-0.2	1.5	125.4	0.8714	-3.9	7.1	43.0	<0.0001
DBA/2J ♀ vs. CAST/EiJ ♂	39.0	11.9	104.3	<0.0001	2.4	7.0	140.0	<0.0001	1.2	8.4	118.4	<0.0001	1.3	2.5	64.5	0.2943
DBA/2J ♀ vs. CAST/EiJ ♀	-21.2	3.6	74.1	0.0218	-5.8	9.3	62.3	<0.0001	0.8	4.5	104.7	0.0006	-5.2	9.0	69.0	<0.0001
DBA/2J ♀ vs. C57Bl/6J ♂	31.7	10.0	96.4	<0.0001	0.2	0.7	159.6	0.9992	1.4	10.3	105.3	<0.0001	-0.3	0.7	62.9	0.9995
DBA/2J ♀ vs. C57Bl/6J ♀	-8.5	2.2	158.4	0.4460	-4.4	10.3	187.1	<0.0001	0.8	5.2	158.4	<0.0001	-4.2	6.9	67.8	<0.0001
DBA/2J ♀ vs. 129S1/SvImJ ♂	32.1	9.8	107.6	<0.0001	-1.5	3.9	181.3	0.0045	1.8	12.9	119.6	<0.0001	0.3	0.5	62.9	>0.9999
DBA/2J ♀ vs. 129S1/SvImJ ♀	23.9	6.7	135.1	<0.0001	-0.7	2.0	218.4	0.5846	1.3	9.6	118.1	<0.0001	-1.9	2.6	58.3	0.2334
DBA/2J ♀ vs. PWD/PhJ ♂	20.0	5.8	125.9	<0.0001	-6.0	18.9	190.6	<0.0001	2.4	18.3	104.8	<0.0001	-6.1	11.6	70.2	<0.0001
DBA/2J ♀ vs. PWD/PhJ ♀	6.7	1.8	142.8	0.7529	-1.3	2.5	122.7	0.2629	0.7	4.1	148.8	0.0023	-2.3	3.4	71.6	0.0361
CAST/EiJ ♂ vs. CAST/EiJ ♀	-60.1	11.3	49.9	<0.0001	-8.2	13.4	56.6	<0.0001	-0.4	2.4	69.4	0.3376	-6.5	14.8	48.2	<0.0001
CAST/EiJ ♂ vs. C57Bl/6J ♂	-7.3	4.3	117.2	0.0015	-2.1	8.1	103.5	<0.0001	0.2	1.9	104.1	0.6758	-1.7	4.9	64.6	0.0003
CAST/EiJ ♂ vs. C57Bl/6J ♀	-47.5	17.3	147.6	<0.0001	-6.7	16.7	152.2	<0.0001	-0.4	3.1	152.1	0.0760	-5.5	11.5	44.4	<0.0001
CAST/EiJ ♂ vs. 129S1/SvImJ ♂	-6.9	3.6	128.8	0.0146	-3.8	11.1	138.1	<0.0001	0.6	5.9	119.4	<0.0001	-1.0	3.0	61.4	0.1004
CAST/EiJ ♂ vs. 129S1/SvImJ ♀	-15.1	6.3	133.2	<0.0001	-3.1	9.2	165.4	<0.0001	0.1	1.3	116.8	0.9406	-3.2	5.1	38.5	0.0004
CAST/EiJ ♂ vs. PWD/PhJ ♂	-19.0	8.9	190.8	<0.0001	-8.3	29.2	130.8	<0.0001	1.2	13.6	103.4	<0.0001	-7.4	20.0	71.6	<0.0001
CAST/EiJ ♂ vs. PWD/PhJ ♀	-32.3	12.1	111.6	<0.0001	-3.6	7.6	105.5	<0.0001	-0.5	3.7	117.3	0.0124	-3.6	6.3	48.2	<0.0001
CAST/EiJ ♀ vs. C57Bl/6J ♂	52.9	10.1	47.7	<0.0001	6.1	10.3	50.3	<0.0001	0.5	3.8	58.0	0.0136	4.8	11.6	47.1	<0.0001
CAST/EiJ ♀ vs. C57Bl/6J ♀	12.6	2.2	63.9	0.4496	1.5	2.2	76.1	0.4355	0.0	0.0	92.5	>0.9999	1.0	1.8	51.0	0.7372
CAST/EiJ ♀ vs. 129S1/SvImJ ♂	53.3	10.0	50.2	<0.0001	4.4	7.0	63.8	<0.0001	1.0	6.4	66.3	<0.0001	5.4	13.0	47.0	<0.0001
CAST/EiJ ♀ vs. 129S1/SvImJ ♀	45.1	8.2	57.3	<0.0001	5.1	8.1	63.0	<0.0001	0.5	3.4	65.7	0.0393	3.2	4.7	45.5	0.0009
CAST/EiJ ♀ vs. PWD/PhJ ♂	41.2	7.6	53.5	<0.0001	-0.1	0.2	53.5	>0.9999	1.6	11.1	57.8	<0.0001	-0.9	2.1	54.8	0.5244
CAST/EiJ ♀ vs. PWD/PhJ ♀	27.9	4.9	62.2	0.0003	4.6	6.4	89.8	<0.0001	-0.1	0.8	99.3	0.9991	2.8	4.6	55.8	0.0010
C57Bl/6J ♂ vs. C57Bl/6J ♀	-40.2	15.4	143.1	<0.0001	-4.6	12.4	145.2	<0.0001	-0.5	4.9	161.5	<0.0001	-3.9	8.3	42.0	<0.0001
C57Bl/6J ♂ vs. 129S1/SvImJ ♂	0.4	0.2	191.2	>0.9999	-1.7	5.5	147.9	<0.0001	0.4	4.9	192.8	<0.0001	0.6	2.0	98.0	0.6207
C57Bl/6J ♂ vs. 129S1/SvImJ ♀	-7.8	3.5	134.5	0.0227	-1.0	3.2	218.2	0.0515	0.0	0.5	187.8	>0.9999	-1.6	2.5	36.1	0.2799
C57Bl/6J ♂ vs. PWD/PhJ ♂	-11.7	6.0	289.0	<0.0001	-6.2	26.0	386.9	<0.0001	1.1	14.3	335.6	<0.0001	-5.8	16.7	102.8	<0.0001
C57Bl/6J ♂ vs. PWD/PhJ ♀	-25.0	9.8	104.0	<0.0001	-1.5	3.3	91.9	0.0468	-0.7	5.4	104.9	<0.0001	-2.0	3.5	45.5	0.0290
C57Bl/6J ♀ vs. 129S1/SvImJ ♂	40.6	14.8	160.2	<0.0001	2.9	6.7	187.4	<0.0001	1.0	8.1	176.3	<0.0001	4.5	9.6	42.2	<0.0001
C57Bl/6J ♀ vs. 129S1/SvImJ ♀	32.5	10.5	186.3	<0.0001	3.6	8.5	204.4	<0.0001	0.5	4.3	173.6	0.0011	2.3	3.2	49.1	0.0709
C57Bl/6J ♀ vs. PWD/PhJ ♂	28.6	9.8	198.0	<0.0001	-1.6	4.2	166.0	0.0017	1.6	14.4	160.8	<0.0001	-1.9	3.9	48.4	0.0097
C57Bl/6J ♀ vs. PWD/PhJ ♀	15.2	4.6	172.6	0.0004	3.1	5.7	151.1	<0.0001	-0.1	0.8	159.4	0.9981	1.9	2.9	58.3	0.1337
129S1/SvImJ ♂ vs. 129S1/SvImJ ♀	-8.2	3.4	151.6	0.0272	0.7	1.9	209.3	0.6437	-0.5	4.8	174.0	0.0001	-2.2	3.5	36.4	0.0336
129S1/SvImJ ♂ vs. PWD/PhJ ♂	-12.1	5.7	257.1	<0.0001	-4.5	13.9	176.2	<0.0001	0.6	7.6	192.8	<0.0001	-6.4	18.3	93.5	<0.0001
129S1/SvImJ ♂ vs. PWD/PhJ ♀	-25.4	9.5	119.6	<0.0001	0.2	0.4	125.7	>0.9999	-1.1	8.2	119.9	<0.0001	-2.6	4.6	45.8	0.0011
129S1/SvImJ ♀ vs. PWD/PhJ ♂	-3.9	1.5	198.5	0.8832	-5.2	16.5	254.7	<0.0001	1.1	13.1	187.7	<0.0001	-4.2	6.5	39.7	<0.0001
129S1/SvImJ ♀ vs. PWD/PhJ ♀	-17.2	5.7	149.6	<0.0001	-0.5	1.0	127.2	0.9887	-0.6	4.8	118.3	0.0002	-0.4	0.5	58.3	>0.9999
PWD/PhJ ♂ vs. PWD/PhJ ♀	-13.3	4.7	149.9	0.0003	4.7	10.2	101.5	<0.0001	-1.7	13.8	104.4	<0.0001	3.8	6.6	50.7	<0.0001
