# Supplementary material for: Chromosome length is not the sole determinant of sexually dimorphic crossover rates during mammalian meiosis: Insights from genetically diverse mouse strains
Source: bioRxiv. 2025 Dec 22:2025.12.19.695521. Preprint. [Version 1] doi: 10.64898/2025.12.19.695521 (PMC12776159; doi:10.64898/2025.12.19.695521)
Supplement: Supplement 8 — B6 female mice were used as a reference group. Odds ratios greater than 1 indicate increased likelihood of an SC having more than one MLH1 focus with a change in the respective variable (increasing SC length or change in strain or sex) while holding all over variables constant. Model fits were evaluated using G2 (likelihood ratio test statistic) and several pseudo-R2 values. Both the main effects only model and the full model (with all interaction terms) showed significantly better fits than the null model (no predictors), and the full model showed marginally better fit than the main effects only model. [file media-8.pdf]

	Estimate	Std. Error	Odds Ratio	CI (low, high)	z-value	p-value
**(Intercept)**	-5.522	0.278	0.004	0.002 , 0.006	-19.849	<0.0001
**SC Length (μm)**	0.522	0.027	1.686	1.599 , 1.78	19.157	<0.0001
**Sex (Male)**	-0.397	0.367	0.672	0.327 , 1.384	-1.082	0.2794
**Strain (129)**	-1.843	0.476	0.158	0.061 , 0.398	-3.875	<0.001
**Strain (CAST)**	1.194	0.451	3.299	1.352 , 7.921	2.650	<0.01
**Strain (DBA)**	1.650	0.386	5.209	2.443 , 11.112	4.273	<0.0001
**Strain (PWD)**	0.301	0.442	1.352	0.565 , 3.199	0.682	0.4951
**SC Length : Sex (Male)**	0.049	0.038	1.050	0.974 , 1.13	1.292	0.1963
**SC Length : Strain (129)**	0.200	0.050	1.221	1.108 , 1.349	3.983	<0.001
**SC Length : Strain (CAST)**	-0.105	0.043	0.900	0.827 , 0.98	-2.434	<0.05
**SC Length : Strain (DBA)**	-0.264	0.037	0.768	0.713 , 0.825	-7.082	<0.0001
**SC Length : Strain (PWD)**	-0.065	0.044	0.937	0.86 , 1.022	-1.462	0.1438
**Sex (Male) : Strain (129)**	1.788	0.621	5.977	1.774 , 20.258	2.879	<0.01
**Sex (Male) : Strain (CAST)**	-2.865	0.770	0.057	0.012 , 0.252	-3.723	<0.001
**Sex (Male) : Strain (DBA)**	-1.989	0.629	0.137	0.039 , 0.464	-3.163	<0.01
**Sex (Male) : Strain (PWD)**	0.378	0.546	1.459	0.502 , 4.268	0.692	0.4888
**SC Length : Sex (Male) : Strain (129)**	-0.134	0.067	0.875	0.766 , 0.997	-1.993	<0.05
**SC Length : Sex (Male) : Strain (CAST)**	0.226	0.080	1.254	1.073 , 1.47	2.819	<0.01
**SC Length : Sex (Male) : Strain (DBA)**	0.173	0.065	1.189	1.046 , 1.351	2.650	<0.01
**SC Length : Sex (Male) : Strain (PWD)**	0.158	0.057	1.171	1.046 , 1.309	2.769	<0.01

Model	log-likelihood	G2	McFadden pseudo-R^2	Cox & Snell's R^2	Nagelkerke's R^2	Chi-square	df	p-value
**Null**	-11770.567	0.000	0.000	0.000	0.000	NA	NA	NA
**Main Effects**	-8767.115	6006.905	0.255	0.280	0.387	6006.90	15	<0.0001
**Full (with Interactions)**	-8752.043	6037.047	0.256	0.281	0.388	30.14	4	<0.0001
