# Supplementary material for: Chromosome length is not the sole determinant of sexually dimorphic crossover rates during mammalian meiosis: Insights from genetically diverse mouse strains
Source: bioRxiv. 2025 Dec 22:2025.12.19.695521. Preprint. [Version 1] doi: 10.64898/2025.12.19.695521 (PMC12776159; doi:10.64898/2025.12.19.695521)
Supplement: Supplement 9 — Both the absolute (microns) and normalized (%SC length) distance between adjacent MLH1 foci on the same SC were analyzed by Kolmogorov-Smirnov (KS) test using pooled data from all SCs, the 5 longest SCs per cell, and the 5 shortest SCs per cell. Top table summarizes median inter-focus distance, and the bottom table summarizes the KS test statistics and their respective Bonferroni-adjusted p-values. [file media-9.pdf]

		Median Interfocus Distance									
	Measurement	DBA/2J ♂	DBA/2J ♀	CAST/EiJ ♂	CAST/EiJ ♀	C57Bl/6J ♂	C57Bl/6J ♀	129S1/SvImJ ♂	129S1/SvImJ ♀	PWD/PhJ ♂	PWD/PhJ ♀
All SCs	Absolute distance (µm)	5.65	5.43	6.15	5.92	6.09	5.79	5.52	5.41	6.14	4.98
	Normalized distance (%SC length)	63.00	51.27	64.27	51.91	64.76	53.50	59.40	55.39	64.74	49.32
Longest 5 SCs	Absolute distance (µm)	6.84	6.67	6.43	6.80	7.02	6.28	6.43	5.85	7.23	5.48
	Normalized distance (%SC length)	65.88	53.82	64.03	49.99	64.62	50.87	59.22	53.18	64.91	48.94
Shortest 5 SCs	Absolute distance (µm)	3.55	2.89	2.54	4.22	4.33	4.38	4.42	3.60	4.76	3.26
	Normalized distance (%SC length)	54.98	41.07	47.73	50.05	64.53	56.34	67.21	46.91	63.17	46.98

	All SCs				Longest 5 SCs				Shortest 5 SCs			
	Absolute Distance (µm)		Normalized Distance (%SC Length)		Absolute Distance (µm)		Normalized Distance (%SC Length)		Absolute Distance (µm)		Normalized Distance (%SC Length)	
Comaprison	KS Stat	Adj. P value	KS Stat	Adj.P value	KS Stat	Adj. P value	KS Stat	Adj.P value	KS Stat	Adj. P value	KS Stat	Adj.P value
DBA ♀ vs. DBA ♂	0.13	1.00	0.26	<0.0001	0.14	1.00	0.32	>0.01	0.38	1.00	0.59	1.00
CAST ♀ vs. CAST ♂	0.12	1.00	0.31	<0.0001	0.22	0.12	0.35	>0.0001	0.40	1.00	0.38	1.00
B6 ♀ vs. B6 ♂	0.09	<0.05	0.34	<0.0001	0.20	<0.0001	0.40	>0.0001	0.14	1.00	0.27	1.00
129 ♀ vs. 129 ♂	0.09	1.00	0.13	<0.05	0.21	<0.0001	0.21	>0.01	0.44	1.00	0.40	1.00
PWD ♀ vs. PWD ♂	0.29	<0.0001	0.45	<0.0001	0.37	<0.0001	0.43	>0.0001	0.46	>0.01	0.49	>0.001
B6 ♀ vs. PWD ♀	0.21	<0.0001	0.15	<0.001	0.19	<0.01	0.10	1.00	0.37	0.53	0.28	1.00
B6 ♀ vs. 129 ♀	0.14	<0.001	0.07	1.00	0.17	<0.05	0.11	1.00	0.39	1.00	0.30	1.00
B6 ♀ vs. CAST ♀	0.09	0.66	0.06	1.00	0.17	0.10	0.07	1.00	0.09	1.00	0.26	1.00
B6 ♀ vs. DBA ♀	0.17	<0.001	0.14	<0.01	0.12	1.00	0.17	0.22	0.42	>0.05	0.44	>0.05
PWD ♀ vs. 129 ♀	0.13	0.08	0.19	<0.0001	0.12	1.00	0.17	0.20	0.16	1.00	0.15	1.00
PWD ♀ vs. CAST ♀	0.22	<0.0001	0.12	0.13	0.29	<0.0001	0.13	1.00	0.33	1.00	0.20	1.00
PWD ♀ vs. DBA ♀	0.15	<0.05	0.13	0.20	0.27	<0.001	0.23	>0.05	0.17	1.00	0.19	1.00
129 ♀ vs. CAST ♀	0.19	<0.0001	0.12	0.10	0.30	<0.0001	0.13	1.00	0.33	1.00	0.20	1.00
129 ♀ vs. DBA ♀	0.13	0.11	0.17	<0.01	0.25	<0.0001	0.13	1.00	0.24	1.00	0.23	1.00
CAST ♀ vs. DBA ♀	0.16	<0.01	0.12	0.35	0.09	1.00	0.12	1.00	0.37	0.18	0.27	1.00
DBA ♂ vs. 129 ♂	0.13	0.93	0.13	1.00	0.21	0.79	0.22	0.34	0.72	0.85	0.61	1.00
DBA ♂ vs. PWD ♂	0.14	0.30	0.13	0.58000	0.11	1.00	0.08	1.00	0.76	0.11	0.72	0.23
DBA ♂ vs. B6 ♂	0.14	0.25	0.14	0.30000	0.06	1.00	0.07	1.00	0.53	1.00	0.57	1.00
DBA ♂ vs. CAST ♂	0.17	0.67	0.10	1.00	0.19	1.00	0.13	1.00	0.40	1.00	0.40	1.00
129 ♂ vs. PWD ♂	0.16	<0.0001	0.16	<0.0001	0.25	<0.0001	0.18	>0.01	0.19	1.00	0.20	1.00
129 ♂ vs. B6 ♂	0.16	<0.0001	0.17	<0.0001	0.20	<0.0001	0.19	>0.01	0.19	1.00	0.16	1.00
129 ♂ vs. CAST ♂	0.19	<0.01	0.15	0.29	0.07	1.00	0.16	1.00	0.49	1.00	0.43	1.00
PWD ♂ vs. B6 ♂	0.04	1.00	0.03	1.00	0.10	1.00	0.06	1.00	0.24	1.00	0.20	1.00
PWD ♂ vs. CAST ♂	0.08	1.00	0.06	1.00	0.23	<0.0001	0.12	1.00	0.54	1.00	0.51	1.00
B6 ♂ vs. CAST ♂	0.06	1.00	0.07	1.00	0.19	0.23	0.11	1.00	0.47	1.00	0.37	1.00
DBA ♀ vs. CAST ♂	0.24	<0.001	0.31	<0.0001	0.20	0.66	0.28	>0.01	0.33	1.00	0.37	1.00
DBA ♀ vs. B6 ♂	0.23	<0.0001	0.36	<0.0001	0.17	0.24	0.31	>0.0001	0.42	0.17	0.50	>0.01
DBA ♀ vs. 129 ♂	0.20	<0.0001	0.25	<0.0001	0.17	0.49	0.23	>0.01	0.49	0.15	0.52	0.06
DBA ♀ vs. PWD ♂	0.23	<0.0001	0.35	<0.0001	0.19	<0.05	0.27	>0.0001	0.52	>0.0001	0.65	>0.0001
CAST ♀ vs. DBA ♂	0.08	1.00	0.26	<0.0001	0.15	1.00	0.39	>0.0001	0.61	1.00	0.43	1.00
CAST ♀ vs. B6 ♂	0.09	1.00	0.36	<0.0001	0.13	1.00	0.38	>0.0001	0.11	1.00	0.37	0.38
CAST ♀ vs. 129 ♂	0.15	<0.001	0.22	<0.0001	0.22	<0.01	0.27	>0.0001	0.16	1.00	0.43	0.42
CAST ♀ vs. PWD ♂	0.09	0.36	0.34	<0.0001	0.10	1.00	0.34	>0.0001	0.19	1.00	0.46	>0.0001
B6 ♀ vs. DBA ♂	0.07	1.00	0.27	<0.0001	0.19	1.00	0.41	>0.0001	0.67	0.74	0.46	1.00
B6 ♀ vs. CAST ♂	0.12	1.00	0.30	<0.0001	0.13	1.00	0.36	>0.0001	0.40	1.00	0.38	1.00
B6 ♀ vs. 129 ♂	0.09	0.16	0.18	<0.0001	0.08	1.00	0.28	>0.0001	0.13	1.00	0.38	1.00
B6 ♀ vs. PWD ♂	0.08	<0.05	0.32	<0.0001	0.21	<0.0001	0.37	>0.0001	0.15	1.00	0.29	0.24
129 ♀ vs. DBA ♂	0.16	0.18	0.24	<0.0001	0.33	<0.0001	0.37	>0.0001	0.36	1.00	0.44	1.00
129 ♀ vs. CAST ♂	0.23	<0.001	0.27	<0.0001	0.23	<0.05	0.31	>0.0001	0.29	1.00	0.29	1.00
129 ♀ vs. B6 ♂	0.22	<0.0001	0.29	<0.0001	0.35	<0.0001	0.34	>0.0001	0.30	1.00	0.38	1.00
129 ♀ vs. PWD ♂	0.22	<0.0001	0.28	<0.0001	0.36	<0.0001	0.33	>0.0001	0.47	0.19	0.49	0.10
PWD ♀ vs. DBA ♂	0.19	<0.05	0.35	<0.0001	0.33	<0.0001	0.49	>0.0001	0.38	1.00	0.46	1.00
PWD ♀ vs. CAST ♂	0.34	<0.0001	0.42	<0.0001	0.30	<0.0001	0.43	>0.0001	0.36	1.00	0.33	1.00
PWD ♀ vs. B6 ♂	0.29	<0.0001	0.46	<0.0001	0.34	<0.0001	0.47	>0.0001	0.32	1.00	0.36	1.00
PWD ♀ vs. 129 ♂	0.18	<0.0001	0.31	<0.0001	0.24	<0.0001	0.36	>0.0001	0.42	1.00	0.44	0.85
