# Supplementary material for: Chromosome length is not the sole determinant of sexually dimorphic crossover rates during mammalian meiosis: Insights from genetically diverse mouse strains
Source: bioRxiv. 2025 Dec 22:2025.12.19.695521. Preprint. [Version 1] doi: 10.64898/2025.12.19.695521 (PMC12776159; doi:10.64898/2025.12.19.695521)
Supplement: Supplement 10 — Both the absolute (microns) and normalized (%SC length) distance between the centromere and the first MLH1 focus for each SC were analyzed by Kolmogorov-Smirnov (KS) test using pooled data from all SCs. The analysis was also performed for SCs with 1 MLH1 focus and multiple MLH1 foci. Top table summarizes the median focus distance from the centromere, and the bottom table summarizes the KS test statistics and their respective Bonferroni-adjusted p-values. [file media-10.pdf]

			Median distance from centromere to first MLH1 focus									
	MLH1 foci per SC		DBA/2J ♂	DBA/2J ♀	CAST/EiJ ♂	CAST/EiJ ♀	C57Bl/6J ♂	C57Bl/6J ♀	129S1/SvImJ ♂	129S1/SvImJ ♀	PWD/PhJ ♂	PWD/PhJ ♀
All SCs	1	Absolute distance (µm)	4.84	4.64	4.83	4.67	5.06	4.70	4.73	4.20	4.83	4.70
		Normalized distance (%SC length)	69.49	53.63	69.29	54.13	70.22	53.77	66.22	56.48	74.06	56.97
	>1	Absolute distance (µm)	2.10	2.53	2.53	2.60	2.24	2.72	2.46	2.36	2.09	2.97
		Normalized distance (%SC length)	23.32	23.70	25.33	23.80	24.14	25.32	26.33	24.74	21.60	29.43

	SCs with 1 MLH1 focus				SCs with >1 MLH1 focus			
	Absolute Distance (µm)		Normalized Distance (%SC Length)		Absolute Distance (µm)		Normalized Distance (%SC Length)	
Comaprison	KS Stat	Adj. P value	KS Stat	Adj.P value	KS Stat	Adj. P value	KS Stat	Adj.P value
DBA ♀ vs. DBA ♂	0.05	1.00	0.21	<0.0001	0.20	<0.05	0.17	0.13
CAST ♀ vs. CAST ♂	0.08	1.00	0.26	<0.0001	0.11	1.00	0.09	1.00
B6 ♀ vs. B6 ♂	0.07	0.05	0.27	<0.0001	0.18	<0.0001	0.09	0.11
129 ♀ vs. 129 ♂	0.13	<0.0001	0.17	<0.0001	0.04	1.00	0.09	1.00
PWD ♀ vs. PWD ♂	0.06	1.00	0.27	<0.0001	0.29	<0.0001	0.26	<0.0001
B6 ♀ vs. PWD ♀	0.04	1.00	0.06	0.90	0.09	0.65	0.15	<0.001
B6 ♀ vs. 129 ♀	0.10	<0.001	0.06	1.00	0.16	<0.0001	0.07	1.00
B6 ♀ vs. CAST ♀	0.03	1.00	0.03	1.00	0.06	1.00	0.08	1.00
B6 ♀ vs. DBA ♀	0.05	1.00	0.07	0.47	0.12	0.10	0.13	<0.05
PWD ♀ vs. 129 ♀	0.10	<0.01	0.04	1.00	0.20	<0.0001	0.19	<0.0001
PWD ♀ vs. CAST ♀	0.06	1.00	0.06	1.00	0.13	<0.05	0.20	<0.0001
PWD ♀ vs. DBA ♀	0.08	0.11	0.09	>0.01	0.16	<0.01	0.22	<0.0001
129 ♀ vs. CAST ♀	0.12	<0.01	0.06	1.00	0.13	0.08	0.06	1.00
129 ♀ vs. DBA ♀	0.11	<0.0001	0.06	1.00	0.10	1.00	0.09	1.00
CAST ♀ vs. DBA ♀	0.05	1.00	0.06	1.00	0.07	1.00	0.12	0.39
DBA ♂ vs. 129 ♂	0.09	<0.01	0.08	<0.05	0.14	0.51	0.14	0.46
DBA ♂ vs. PWD ♂	0.06	1.00	0.08	<0.0001	0.06	1.00	0.10	1.00
DBA ♂ vs. B6 ♂	0.05	0.45	0.08	<0.0001	0.07	1.00	0.07	1.00
DBA ♂ vs. CAST ♂	0.09	<0.05	0.07	1.00	0.18	0.53	0.13	1.00
129 ♂ vs. PWD ♂	0.05	1.00	0.15	<0.0001	0.14	<0.0001	0.15	<0.0001
129 ♂ vs. B6 ♂	0.09	<0.001	0.11	<0.0001	0.08	0.77	0.09	0.22
129 ♂ vs. CAST ♂	0.04	1.00	0.10	<0.01	0.05	1.00	0.05	1.00
PWD ♂ vs. B6 ♂	0.06	0.12	0.06	0.16	0.08	0.06	0.09	0.01
PWD ♂ vs. CAST ♂	0.04	1.00	0.07	0.39	0.16	<0.05	0.15	0.15
B6 ♂ vs. CAST ♂	0.06	1.00	0.05	1.00	0.11	1.00	0.10	1.00
DBA ♀ vs. CAST ♂	0.11	<0.001	0.27	<0.0001	0.11	1.00	0.14	1.00
DBA ♀ vs. B6 ♂	0.09	<0.0001	0.27	<0.0001	0.13	<0.05	0.14	>0.05
DBA ♀ vs. 129 ♂	0.10	<0.01	0.21	<0.0001	0.10	1.00	0.14	>0.05
DBA ♀ vs. PWD ♂	0.08	<0.05	0.33	<0.0001	0.15	<0.001	0.13	>0.01
CAST ♀ vs. DBA ♂	0.04	1.00	0.22	<0.0001	0.22	<0.0001	0.09	1.00
CAST ♀ vs. B6 ♂	0.08	0.74	0.23	<0.0001	0.16	<0.0001	0.05	1.00
CAST ♀ vs. 129 ♂	0.10	0.26	0.20	<0.0001	0.12	<0.05	0.10	0.48
CAST ♀ vs. PWD ♂	0.06	1.00	0.27	<0.0001	0.18	<0.0001	0.08	1.00
B6 ♀ vs. DBA ♂	0.12	<0.0001	0.26	<0.0001	0.24	<0.0001	0.14	0.41
B6 ♀ vs. CAST ♂	0.14	<0.0001	0.25	<0.0001	0.12	1.00	0.05	1.00
B6 ♀ vs. 129 ♂	0.16	<0.0001	0.22	<0.0001	0.14	<0.0001	0.04	1.00
B6 ♀ vs. PWD ♂	0.14	<0.0001	0.32	<0.0001	0.21	<0.0001	0.14	>0.0001
129 ♀ vs. DBA ♂	0.03	1.00	0.21	<0.0001	0.12	1.00	0.13	1.00
129 ♀ vs. CAST ♂	0.07	0.74	0.22	<0.0001	0.07	1.00	0.07	1.00
129 ♀ vs. B6 ♂	0.08	0.07	0.22	<0.0001	0.06	1.00	0.08	1.00
129 ♀ vs. PWD ♂	0.05	1.00	0.28	<0.0001	0.11	<0.05	0.12	>0.01
PWD ♀ vs. DBA ♂	0.05	1.00	0.21	<0.0001	0.31	<0.0001	0.23	>0.001
PWD ♀ vs. CAST ♂	0.07	1.00	0.21	<0.0001	0.18	0.08	0.15	0.49
PWD ♀ vs. B6 ♂	0.09	<0.01	0.22	<0.0001	0.24	<0.0001	0.19	>0.0001
PWD ♀ vs. 129 ♂	0.07	0.49	0.16	<0.0001	0.19	<0.0001	0.12	>0.05
