# Supplementary material for: Chromosome length is not the sole determinant of sexually dimorphic crossover rates during mammalian meiosis: Insights from genetically diverse mouse strains
Source: bioRxiv. 2025 Dec 22:2025.12.19.695521. Preprint. [Version 1] doi: 10.64898/2025.12.19.695521 (PMC12776159; doi:10.64898/2025.12.19.695521)
Supplement: Supplement 11 — Table S5 List of antibodies used in immunofluorescence staining experiments [file media-11.pdf]

Antibody	Dilution Used	Vendor	Catalog Number
Mouse anti-MLH1 mAb	1:100	BD Biosciences	550838
Guinea pig anti-MLH3 pAb	1:500	Custom made with Thermo-Fisher	Horan et al., 2024
Rabbit anti-RAD51 pAb	1:500	Millipore	PC130
Rabbit anti-MSH4 pAb	1:100	ABclonal	A8556
Rabbit anti-CCNB1IP1	1:100	ABclonal	A16693
Mouse anti-SYCP3 mAb	1:1,000	Abcam	ab97672
Rabbit anti-SYCP3 pAb	1:10,000	Custome made	Kolas et al., 2005
Human anti-Centromere Protein pAb	1:1,000	Antibodies Inc.	15-234
Alexa Fluor® 488 AffiniPure® $F(ab')_2$ Fragment Goat Anti-Mouse IgG, Fcγ fragment specific	1:1,000	Jackson ImmunoResearch	115-546-008
Rhodamine Red™-X (RRX) AffiniPure® $F(ab')_2$ Fragment Goat Anti-Mouse IgG, Fcγ fragment specific	1:1,000	Jackson ImmunoResearch	115-296-071
Alexa Fluor® 488 AffiniPure® $F(ab')_2$ Fragment Goat Anti-Rabbit IgG, Fc fragment specific	1:1,000	Jackson ImmunoResearch	111-546-046
Rhodamine Red™-X (RRX) AffiniPure® $F(ab')_2$ Fragment Goat Anti-Rabbit IgG, Fc fragment specific	1:1,000	Jackson ImmunoResearch	111-296-046
Alexa Fluor® 647 AffiniPure® $F(ab')_2$ Fragment Goat Anti-Guinea Pig IgG (H+L)	1:1,000	Jackson ImmunoResearch	106-606-003
Alexa Fluor® 647 AffiniPure® $F(ab')_2$ Fragment Goat Anti-Human IgG, Fcγ fragment specific	1:1,000	Jackson ImmunoResearch	109-606-170
