# Supplementary material for: Chromosome length is not the sole determinant of sexually dimorphic crossover rates during mammalian meiosis: Insights from genetically diverse mouse strains
Source: bioRxiv. 2025 Dec 22:2025.12.19.695521. Preprint. [Version 1] doi: 10.64898/2025.12.19.695521 (PMC12776159; doi:10.64898/2025.12.19.695521)
Supplement: Supplement 1 — Representative images of spermatocytes and oocytes for DBA (A-D), CAST (E-H), B6 (I-L), 129S1 (M-P), and PWD (Q-T) mice. (A-B, E-F, I-J, M-N, and Q-R) pachytene chromosome spreads stained for MLH1 (class I COs, green), SYCP3 (chromosome axis, magenta), and CREST (centromeres, blue). (C-D, G-H, K-L, O-P, S-T) Giemsa-stained chiasmata spreads from diakinesis/metaphase I chromosome spreads. Scale bars represent 10 µm. [file media-1.pdf]

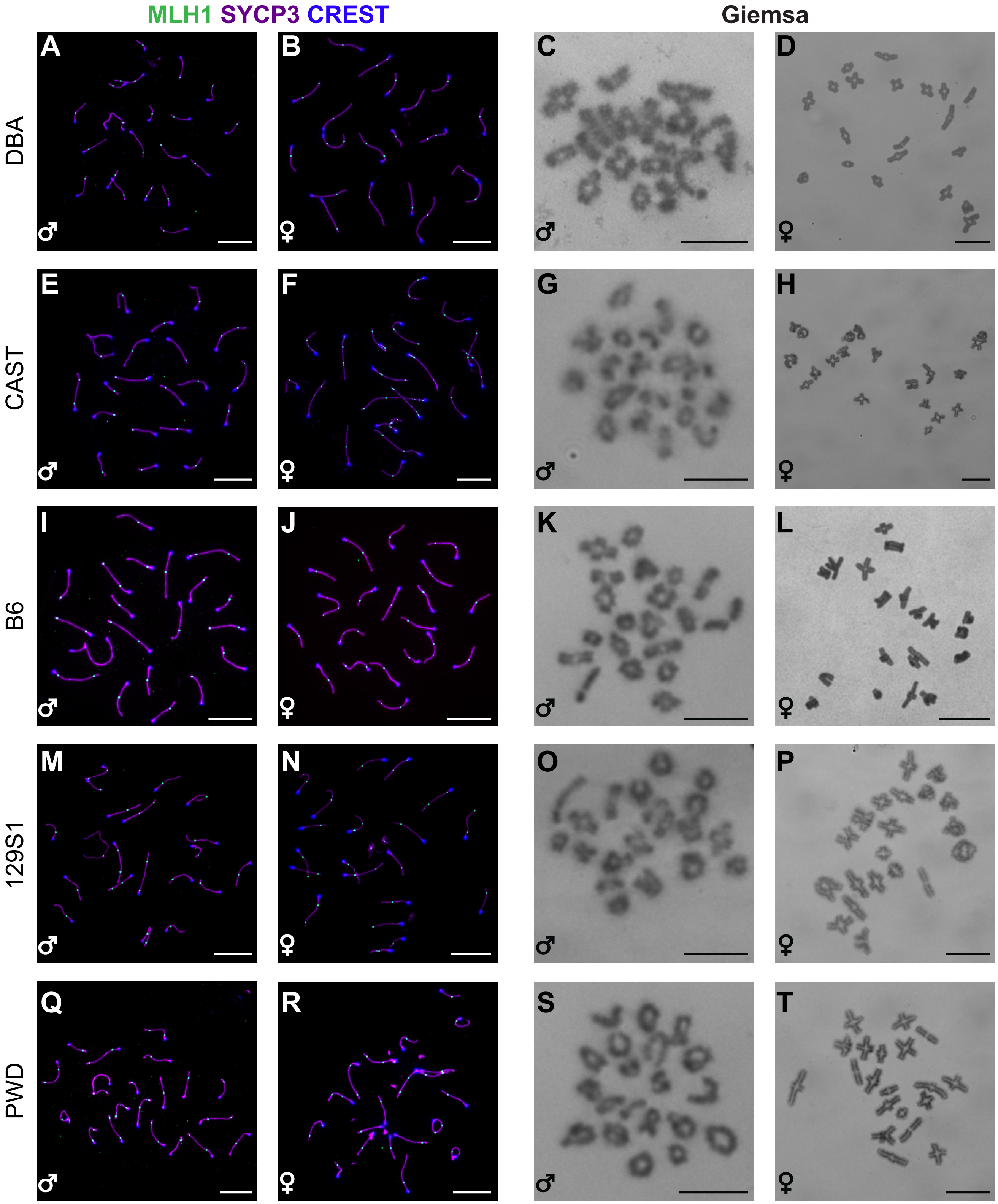
MLH1 SYCP3 CREST
Giemsa
DBA
CAST
B6
129S1
PWD
A
B
C
D
E
F
G
H
I
J
K
L
M
N
O
P
Q
R
S
T
♂
♀
